# Mycoreovirus genome rearrangements associated with RNA silencing deficiency

**DOI:** 10.1093/nar/gkv239

**Published:** 2015-03-23

**Authors:** Ana Eusebio-Cope, Nobuhiro Suzuki

**Affiliations:** Agrivirology Laboratory, Institute of Plant Science and Resources, Okayama University, Kurashiki, Okayama 710–0046, Japan

## Abstract

Mycoreovirus 1 (MyRV1) has 11 double-stranded RNA genome segments (S1 to S11) and confers hypovirulence to the chestnut blight fungus, *Cryphonectria parasitica*. MyRV1 genome rearrangements are frequently generated by a multifunctional protein, p29, encoded by a positive-strand RNA virus, Cryphonectria hypovirus 1. One of its functional roles is RNA silencing suppression. Here, we explored a possible link between MyRV1 genome rearrangements and the host RNA silencing pathway using wild-type (WT) and mutant strains of both MyRV1 and the host fungus. Host strains included deletion mutants of RNA silencing components such as dicer-like (dcl) and argonaute-like (agl) genes, while virus strains included an S4 internal deletion mutant MyRV1/S4ss. Consequently, intragenic rearrangements with nearly complete duplication of the three largest segments, i.e. S1, S2 and S3, were observed even more frequently in the RNA silencing-deficient strains Δ*dcl2* and Δ*agl2* infected with MyRV1/S4ss, but not with any other viral/host strain combinations. An interesting difference was noted between genome rearrangement events in the two host strains, i.e. generation of the rearrangement required prolonged culture for Δ*agl2* in comparison with Δ*dcl2*. These results suggest a role for RNA silencing that suppresses genome rearrangements of a dsRNA virus.

## INTRODUCTION

Generally, RNA viruses are prone to recombination, which is one of the driving forces of virus molecular diversity and evolution (1–5). Two distinct mechanisms of RNA recombination have been proposed, i.e. RNA replicase-dependent (template switch) and -independent (also known as breakage and rejoining) recombination (6). The first category includes several variants. Some of them may require similarities of sequence between the donor, acceptor and/or nascent RNAs, direct and/or indirect repeats in them, or specific secondary structural features (3,7–10). RNA recombinants have long been utilized for studies of viral RNA replication, which have led to the identification of RNA sequences and protein elements important for RNA synthesis, virion assembly and symptom expression. This is particularly true for members of the family *Reoviridae*, where reverse genetics is mostly unavailable (11,12). The *Reoviridae* is one of the largest families of double-stranded (ds) RNA viruses and includes members infecting a wide range of eukaryotic organisms (fungi, plants and animals). Rearrangement events have been reported for all the major genera, characterized by large deletions and/or extensions that are frequently accompanied by substitutions (13). The development of reverse genetics for animal reoviruses such as orthoreoviruses (helper virus-independent) (14) and rotaviruses (helper virus-dependent) (15–17) has led to the creation of artificial replicable genome segments that retain authentic essential terminal sequences (18,19).

RNA silencing or RNA interference (RNAi) is an RNA-mediated post-transcriptional gene regulation mechanism conserved in eukaryotic organisms (20,21). In general, long dsRNA or highly structured single-stranded (ss) RNA molecules are recognized and digested by an RNase III-like endonuclease called Dicer or Dicer-like (DCL) protein into small interfering (si) dsRNAs with a length of 21–26 nucleotides (nt) (dicing). One of the strands incorporated into the RNA silencing induced silencing complex (RISC) guides it to its targets. A major component of RISC, Argonaute or Argonaute-like (AGL) protein mediates target RNA cleavage (slicing). Another major player is RNA-dependent RNA polymerase (RDR), a gene involved in the amplification and transitive spreading of siRNAs.

The RNA mycoviruses/filamentous fungi system provides a unique opportunity to investigate RNA recombination associated with RNA silencing (22). Nuss and co-workers showed that RNA silencing components such as DCL2 and AGL2 are essential for the generation and/or maintenance of defective-interfering (DI) RNA molecules of a positive-sense (+) ssRNA virus, Cryphonectria hypovirus 1 (CHV1), in *Cryphonectria parasitica* (chestnut blight fungus) (23,24). That is, in RNA silencing-defective fungal strains lacking either *dcl2* (Δ*dcl2*) or *agl2* (Δ*agl2*) CHV1 DI-RNA does not occur, whereas it is present spontaneously and frequently appear in RNA silencing-competent strains. While *C. parasitica* has four RDR gene homologues, no phenotypic effects result from the disruption of any these genes (*rdr1–4*) (25). Furthermore, CHV1 vector constructs (artificial recombinants) can retain foreign inserts more stably in RNA silencing-defective *C. parasitica* mutants than in the parental wild type (WT) strain (24).

Mycoreoviruses (genus *Mycoreovirus*, family *Reoviridae*) undergo distinct genome rearrangements as well as the usual ones similar to those found in other *Reoviridae* members (13). Mycoreovirus 1 (MyRV1), with 11 dsRNA genome segments (S1–S11, each encoding single proteins VP1–VP11), infects *C. parasitica* and attenuates its virulence, similarly to CHV1 (26–28). Co-infection of the fungal host with CHV1 and MyRV1 results in a one-way interaction from CHV1 to MyRV1 (trans-enhancement of MyRV1 replication but not CHV1 replication) and frequent generation of MyRV1 rearrangements (29–31). This one-way synergism and rearrangement inductions are phenocopied by transgenic expression of a multi-functional protein of CHV1, p29, in *C. parasitica*. Different types of rearrangements have been identified in CHV1-p29 transformants, with the rearranged genome segments being designated S1L, S2L, S3L, S6L and S10ss (segment numbers indicate their origins, L refers to an extension and ss refers to a long deletion). Most of them are stably maintained in p29 transformant strains. Functional roles assigned to CHV1 p29 include activities as an RNA silencing suppressor, an essential N-terminal *cis*-element for translation and/or RNA synthesis, a papain-like protease, a symptom determinant and a replication enhancer of homologous and heterologous viruses (29,32–36). Among these activities, the protease activity is not required for promotion of MyRV1 rearrangements (L. Sun and N. Suzuki, unpublished results). Thus, MyRV1 genome rearrangement is suspected to occur in concert with p29 functions probably related to RNA silencing suppression and/or enhanced virus replication. Independently of p29, genome rearranged strains MyRV1/S4ss and MyRV1/S10ss, containing a three-fourths internal deletion in the S4 (VP4) and S10 (VP10) open reading frames (ORFs), have been isolated infrequently (12). During an extended study of MyRV1 rearrangements, we found that MyRV1/S4ss frequently underwent further rearrangements in p29 transformants.

In the present study, we addressed whether there is a link between MyRV1 rearrangements and RNA silencing, using a suite of rearranged variant MyRV1 and mutant *C. parasitica* strains whose RNA silencing components had been knocked out. Interestingly, in the RNA silencing-defective fungal strains Δ*dcl2* and Δ*agl2*, MyRV1/S4ss, but not the WT virus, was shown to undergo further rearrangements. Moreover, rearrangements were observed in Δ*dcl2* faster than in Δ*agl2*. Finally, a molecular mechanism underlying MyRV1 genome rearrangements was proposed on the basis of these results.

## MATERIALS AND METHODS

### Viral and fungal strains, their maintenance and inoculation

The viral and fungal strains used in this study are listed in Table 1. The WT virus strain MyRV1 and its variant MyRV1/S4ss carrying the rearranged segment S4ss (internal deletion at the positions 225–2034 nt) and lacking normal S4 have been described previously (12,26,27).

**Table 1.** Viral and fungal strains used in this study

Strain	Description	Reference or Source
***Viral***		
MyRV1	Prototype of the genus *Mycoreovirus*	Suzuki *et al*. (2004)
MyRV1/S10ss	MyRV1 carrying an S10ss variant (S10ss2) with a large internal deletion but lacking normal S10	Sun & Suzuki (2008)
MyRV1/S4ss	MyRV1 carrying an S4ss variant (S4ss3) with a large internal deletion but lacking normal S4	Eusebio-Cope *et al*. (2010)
MyRV1/S4ss+DLSs	MyRV1 carrying S4ss3 plus duplicated large segments (DLSs; S1D, S2D and S3D)	This study
***Fungal***		
EP155	Virus-free field isolate of *C. parasitica*	ATCC 38755
EP155/MyRV1	EP155 infected with wild-type MyRV1	This study
EP155/MyRV1/S4ss	EP155 infected with a rearranged strain MyRV1/S4ss3	This study
Δ*dcl1*	*dcl1* knock-out mutant of EP155	Segers *et al*. (2007)
Δ*dcl2*	*dcl2* knock-out mutant of EP155	Segers *et al*. (2007)
Δ*agl2*	*agl2* knock-out mutant of EP155	Sun *et al*. (2009)
Δ*rdr1*	*rdr1* knock-out mutant of EP155	This study
Twtp29	EP155 transformed with the p29 coding domain	Sun *et al*. (2006)

The genes encoding possible key enzymes for RNA silencing in the fungus are two DCL (*dcl1* and *dcl2*), four AGL (*agl1* to *agl4*) and four RDR (*rdr1* to *rdr4*) genes (23,25,37,38). Fungal cultures of WT *C. parasitica* (EP155) or its mutants lacking the genes for *dcl1* (Δ*dcl1*), *dcl2* (Δ*dcl2*) and *agl2* (Δ*agl2*) were generous gifts from Dr. Donald L. Nuss (23,37). An *rdr1* disruptant was prepared in this study as described below.

Transgenic EP155 containing the coding domain of CHV1-EP713 p29 (Twtp29) has been reported earlier (29,30).

### Hyphal anastomosis, generation of rearranged genomic dsRNA segments and their maintenance

Hyphal anastomosis was carried out between fungal strains which were vegetatively compatible, aiming to horizontally transmit MyRV1. Prior to anastomosis, the fungal partners were refreshed in potato dextrose agar (PDA) for at least 5–7 days. Each anastomosing fungal pair was inoculated in close proximity (10 mm) to each other in a 10-cm plate containing 25 ml of PDA (co-culturing) and three replications were performed. Mycelial blocks were taken randomly from the fungal pair, i.e. the donor (virus source) side and the recipient side (virus-free strain) to check the movement of the virus and eventual gene rearrangement. The plates were incubated at room temperature for approximately 4–5 weeks for the standard procedure (Supplementary Figure S1), or as specified.

For stability and rearranged segment biogenesis assays (for details, see the Results section), after four weeks of incubation, each virus strain was transferred to EP155, Δ*dcl2* and/or Δ*agl2* hosts under the same conditions simultaneously and maintained for the same period. Newly MyRV1-acquired recipient fungi in the first transmission experiment were used as the virus donor for the second co-culture, as well as for the third co-culture.

### Extraction of double-stranded and single stranded RNA

Quick and small-scale dsRNA preparation in 2.0 ml and 1.5 ml Eppendorf tubes was carried out. Briefly, mycelial colonies grown for 3–5 days were peeled off from a PDA plate lined with cellophane, extracted with STE buffer (10 mM Tris-HCl pH 8, 1 mM EDTA and 150 mM NaCl) plus 1.2% SDS, and clarified with one round each of phenol-chloroform and chloroform. The resulting aqueous layer was mixed with a 15% volume of ethanol (EtOH) and a small amount (0.1 g) of CC41 cellulose (Whatman) for column chromatography of dsRNAs. After 1–2 h of continuous agitation at room temperature, each tube was washed with STE-ethanol (15% EtOH; v/v) three times with brief vortexing and centrifugation between washes. The bound dsRNA was eluted once or twice from dried CC41 powder by the addition of STE buffer and precipitated with ethanol. Each sample was subjected to 1% or 1.4% agarose gel electrophoresis and assessed for the production of rearranged genomic dsRNA segments.

Preparation of single-stranded RNA for northern blotting followed the procedure of Suzuki and Nuss (39). Briefly, mycelial blocks of *C. parasitica* strains were inoculated in 50 ml PDB and extracted in two rounds of phenol-chloroform followed by one round of chloroform. After precipitation of the resulting nucleic acid in EtOH, RNA was treated with RQ1 RNase-free DNase I (Promega) and an RNase inhibitor. The single-stranded RNA preparation was enriched by precipitation with 2 M LiCl. Washing, pre-hybridization and hybridization were carried out as described previously (12,30). The probing and detection steps were performed as recommended by the manufacturer (Roche Diagnostics).

### RT-PCR and sequence analysis

For each rearranged strain, cDNA was prepared following the reverse transcriptase-polymerase chain reaction (RT-PCR) conditions set out by Sun and Suzuki (30). Clean preparations of dsRNA from rearranged strains were reverse-transcribed using Moloney murine leukemia virus reverse transcriptase (Fermentas) after denaturing of the sample in 90% dimethylsulfoxide (DMSO) for 15 min at 65°C (40). Portions of rearranged MyRV1 segments were amplified by RT-PCR with different primer sets, and cloned into pGEM-T Easy (Promega), after which their sequences were determined.

Semi-quantitative RT-PCR was similarly employed to confirm the presence and type of the rearranged segments generated during the transmission experiments.

The primers used in this study are listed in Supplementary Table S1.

## RESULTS

### Disruption of *rdr1* in *C. parasitica*

A knockout mutant for the *rdr1* gene (Δ*rdr1*) was generated by homology-dependent gene replacement technology by N. Suzuki in the Nuss laboratory. One of the RDR homologs, *rdr1*, is an orthologous gene to *Neurospora crassa qde1*, a major player in quelling (RNA silencing in *N. crassa*) (41). We made an *rdr1*-knockout construct for homology-dependent gene replacement (Supplementary Figure S2 A; sequence available upon request). After screening of approximately 50 transformants of the WT fungal strain (EP155) with the construct, a disruptant strain (Δ*rdr1*) was obtained. Disruption of the *rdr1* gene was confirmed by Southern blot and RT-PCR analyses (Supplementary Figure S2 B and C). Namely, the *rdr1* gene was replaced with a gene cassette encoding hygromycin B phosphotransferase (*hph*), which confers resistance to hygromycin; neither of the *rdr1*-specific DNA bands was detected by Southern blotting or RT-PCR analysis (see Supplementary Table S1 for primer sequences). Positive reactions were found with the WT fungus (EP155). Note that Δ*rdr1* was prepared independently of the *rdr1* disruptants reported recently by (25).

### Duplicated large segments are generated in RNA silencing-defective fungi infected with MyRV1/S4ss

Since RNA silencing is anticipated to be one of the most important factors affecting MyRV1-genome rearrangement (see the Introduction), we examined this possibility using WT and mutant *C. parasitica* strains (Table 1). The WT virus (MyRV1) and a rearranged variant MyRV1/S4ss3 (12) (Supplementary Figure S3) were introduced via hyphal anastomosis (horizontal virus transmission by co-culturing; see Materials and Methods) from a virus donor strain, EP155, infected with the respective virus strain, into a recipient strain, i.e. a mutant lacking the RNA silencing component Δ*dcl1*, Δ*dcl2*, Δ*rdr1* or Δ*agl2*. S4ss3 is abbreviated to S4ss here. The transgenic EP155 fungal host carrying a CHV1 p29-encoding domain (Twtp29), a potent inducer of rearrangements, was also included in the assay (Table 2).

**Table 2.** Frequency (%) of rearrangement occurrence in different fungus/virus combinations

Host^a^	Virus	Experiment number				
		1	2	3	4	5
EP155	MyRV1 (WT)	0/54	0/72	0/18	0/10	-
	MyRV1/S4ss	0/54	0/72	0/18	0/10	-
Δ*dcl1*	MyRV1 (WT)	0/54	-	-	-	-
	MyRV1/S4ss	0/54	-	-	-	-
Δ*dcl2*	MyRV1 (WT)	0/54	0/72	0/18	0/10	0/50
	MyRV1/S4ss	40/54 (74%)	58/72 (81%)	18/18 (100%)	20/20 (100%)	35/50 (70%)
Δ*agl2*	MyRV1 (WT)	-	-	-	0/10	0/50
	MyRV1/S4ss	-	-	-	8/30 (27%)	3/50 (6%)
Δ*rdr1*	MyRV1 (WT)	-	-	0/18	0/10	-
	MyRV1/S4ss	-	-	0/18	0/10	-
Twtp29	MyRV1 (WT)	11/54 (20%)	-	-	-	-
	MyRV1/S4ss	10/54 (19%)	-	-	-	-
Total		432	288	108	110	200

^a^EP155, Δ*dcl1*, Δ*dcl2*, Δ*rdr1*, Δ*agl2* and Twtp29 refer to the *Cryphonectria parasitica* wild-type strain, its mutant strains lacking *dcl1, dcl2, rdr1* and *agl2*, and the transgenic strain expressing p29 encoded by Cryphonectria hypovirus 1. Frequency is presented as the ratio of subcultures carrying further rearranged segments to the subcultures tested.

**Figure 1.**
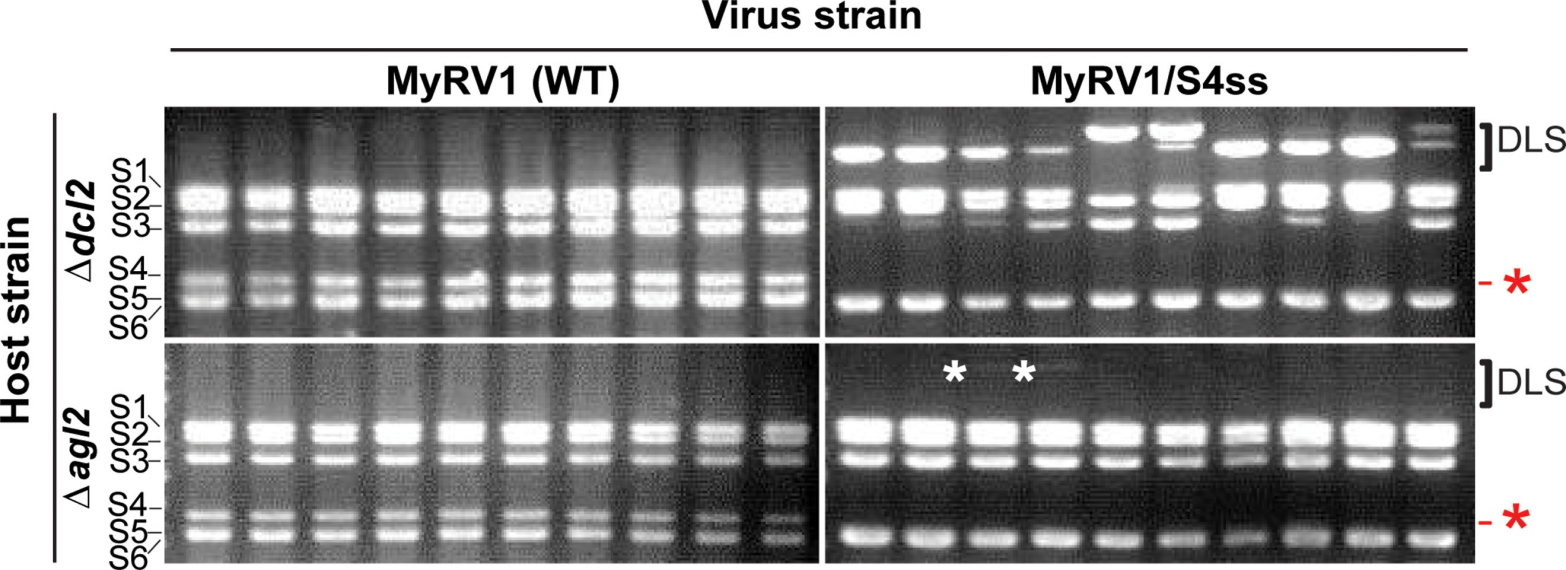
Generation of duplicated large segments (DLSs) in RNA silencing-deficient strains of *Cryphonectria parasitica* infected by MyRV1/S4ss. DsRNA fractions were obtained at 40 days a.c. from *C. parasitica* EP155, Δ*dcl2* and Δ*agl2* infected by WT MyRV1 and MyRV1/S4ss, and electrophoresed on a 1.4% agarose gel. DLSs indicated by brackets are visible above the largest MyRV1 segment S1 in MyRV1/S4ss-infected Δ*dcl2* but are less obvious in MyRV1/S4ss-infected Δ*agl2* (DLSs indicated by white asterisks). No DLSs were detected in the WT EP155 strain infected with either virus (data not shown), WT MyRV1-infected Δ*dcl2* or WT MyRV1-infected Δ*agl2*. The absence (red asterisk) of S4 and presence of S4ss in MyRV1/S4ss samples are denoted on the right. Migration positions of the MyRV1 genome segments are shown on the left in this and in subsequent figures.

Standard rearrangement assays (Supplementary Figure S1) showed that duplicated large segments (DLSs) of different sizes and intensities with slower migration than S1 (4127 bp) in agarose gel electrophoresis were observed approximately 40 days after co-culturing (a.c.) at a frequency of 70–100% (Table 2, experiments 1–5) when MyRV1/S4ss was introduced into the Δ*dcl2* fungal host. Some Δ*dcl2* sub-isolates contained multiple DLSs. In a similar manner, Δ*agl2*, which received MyRV1/S4ss, also potentiated genome rearrangement, albeit at a low frequency (6–27%) (Table 2, experiments 4 and 5). Twtp29 gave a frequency similar to Δ*agl2* for DLS production in MyRV1/S4ss and WT MyRV1 (19–20%; Table 2, experiment 1). However, no rearrangement of the WT MyRV1 genome was found in any of the tested host strains, except for Twtp29. Representative dsRNA gel profiles of the DLS-carrying variants, produced in MyRV1/S4ss-infected Δ*dcl2*, are shown in Figure 1. Note that the DLSs in Δ*agl2* showed faint band intensities (Figure 1). The colony morphologies of the host strains infected with MyRV1 and MyRV1/S4ss are shown in Supplementary Figure S4. It was noted that MyRV1/S4ss induced slower growth rates regardless of the host strain, as reported by (12).

Taken together, MyRV1/S4ss was shown to undergo further rearrangement characterized by the generation of DLSs in Twtp29, Δ*dcl2* and Δ*agl2*, but not in WT EP155, Δ*dcl1* and Δ*rdr1*, where antiviral-defense is fully competent (Table 2). No obvious rearrangement of WT MyRV1 was observed in any fungal host strain except for Twtp29. Although the Twtp29 strains infected with MyRV1 and MyRV1/S4ss showed similar frequencies of rearrangements (20% and 19%), their profiles were different. Most of the rearrangements in the former strain were S10ss, as reported earlier (30), while those in the second were largely DLSs (Supplementary Figure S5, Table S2).

### Duplicated large segments are extended versions of the three largest segments of MyRV1 S1, S2 and S3

**Figure 2.**
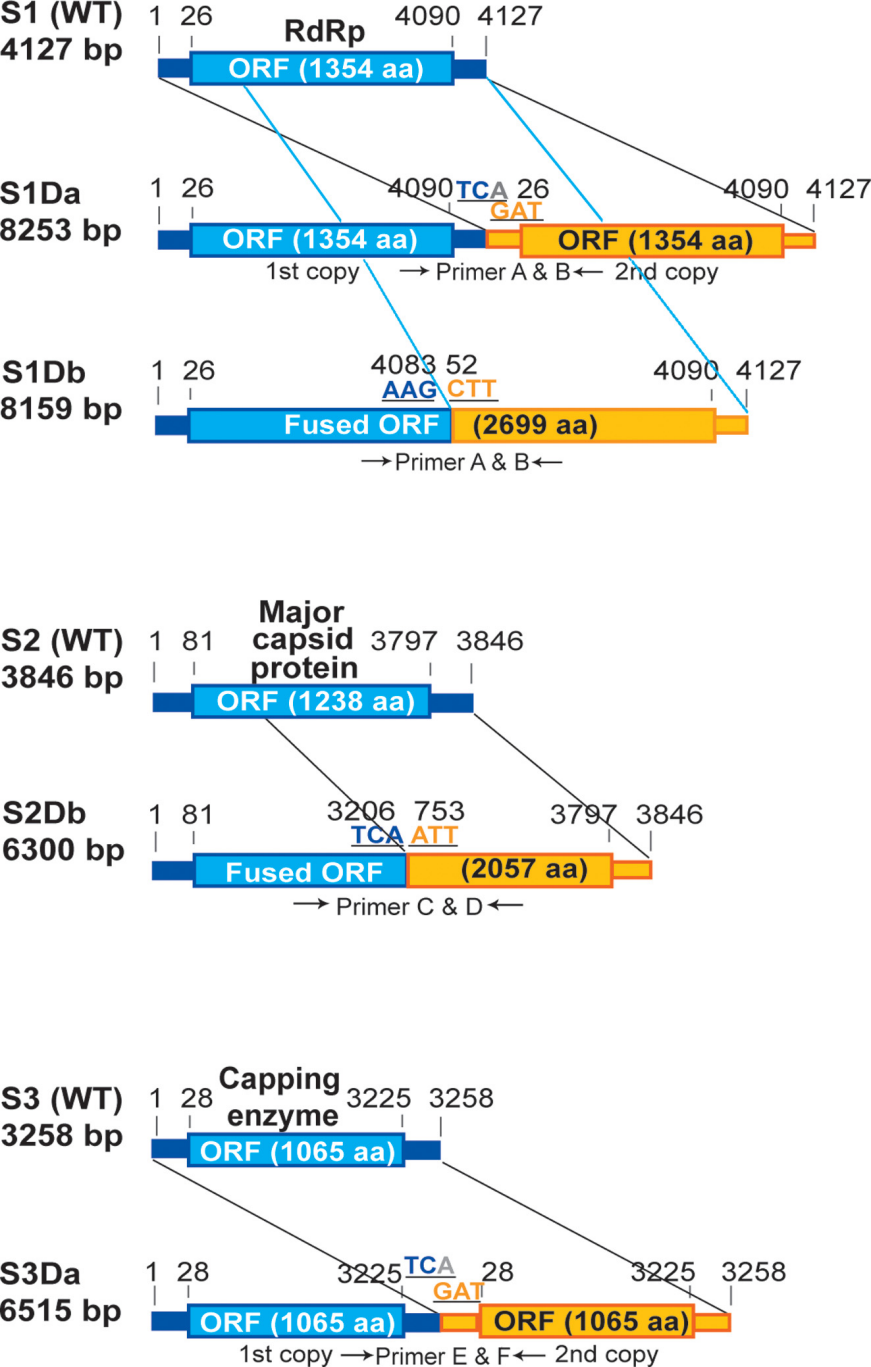
Genome organization of WT MyRV1 segments S1–S3 and their corresponding DLSs. Thick bars represent non-coding regions, while blue and yellow boxes indicate coding domains of the original and extended segments. The rearranged segment of S1 shows two variants, a head to tail duplication (a type, S1Da), and an in-frame duplication of the S1 sequence (b type, S1Db). The rearranged segments for S2 (b type, S2Db) and S3 (a type, S3Da) have a variant with an in-frame duplication or a head to tail duplication, respectively. Nucleotide positions of the start and stop codons and the extension junctions are indicated. DLS lengths are presented on the left in parallel with cognate segments. Primer positions used for sequence determination are indicated by arrows below the DLSs.

The appearance of DLSs was accompanied by the loss of a particular segment or reduced intensity of a particular segment that corresponded to one of MyRV1 S1, S2 or S3 in the agarose gel (Figure 1). Therefore, based on the dsRNA-banding patterns of MyRV1/S4ss with DLSs, it was anticipated that DLSs were derived from S1 to S3. Representative MyRV1 variants stably carrying DLSs originating from S1, S2 and S3 were selected for sequence analyses. Figure 2 shows a diagram representing the genetic organization of the selected DLSs. Two forms of rearrangements in S1 occurred: one was a head-to-tail duplication (a type) of almost the entire genome sequence (S1Da), similar to the human rotavirus rearranged segment 7 (10), while the second type (S1Db) was truncated at nt position 4083 and connected in-frame (b type) at nt position 52 and would encode duplicated VP1. The rearrangement observed for S2Db followed that of the S1Db type, also producing an extended ORF 1.7-fold larger than the original S2 ORF. Similarly to S1Da, the rearranged S3Da had a duplication of the S3 sequence, the first copy lacking the extreme 3′ end nucleotide. All of these rearrangements were much larger than those recently reported for the p29-mediated rearrangements S1L, S2L and S3L (31). In this study, we failed to detect rearranged segments with extensions derived from other segments such as S6, for which an extension rearrangement has been reported (30).

The sequences of all rearranged segments reported here have been deposited in the DDBJ/EMBL/GenBank databases under accession nos. LC019123-LC019126.

### Duplicated large segments appear more rapidly in Δ*dcl2* than in Δ*agl2* upon receipt of MyRV1/S4ss

**Figure 3.**
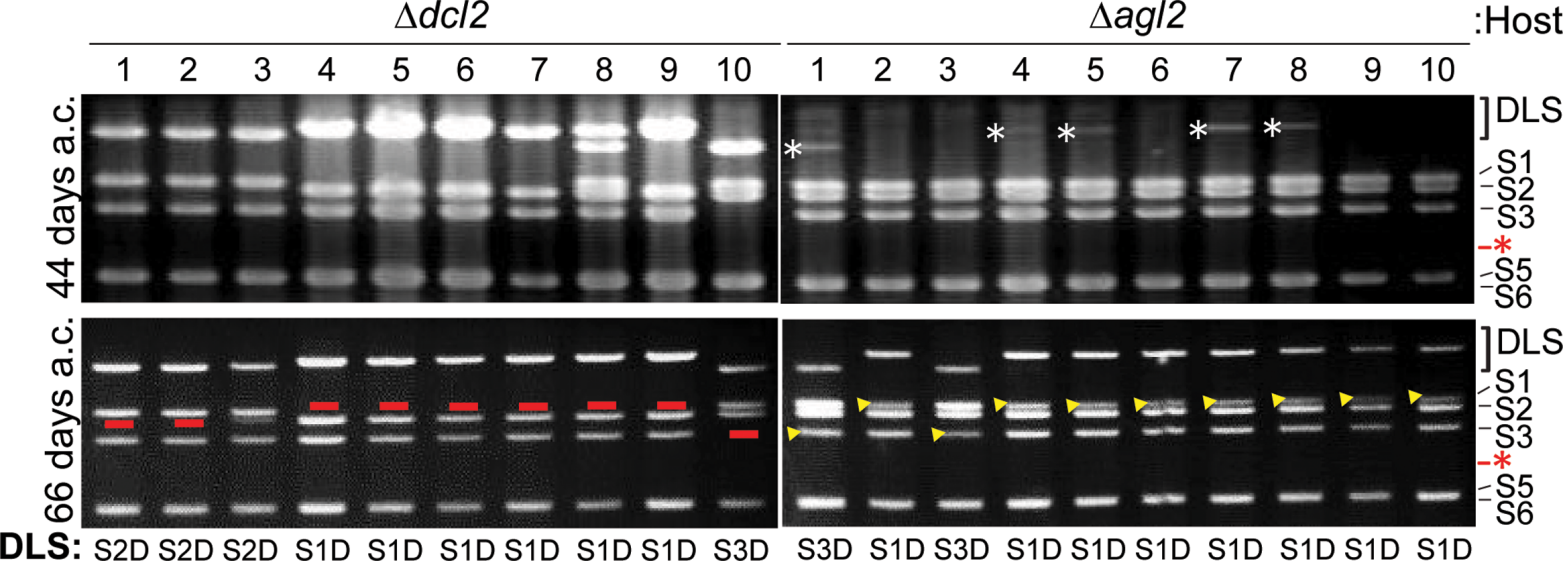
Differences in DLS-generation profiles between the two RNA-silencing deficient hosts. Agarose gel electrophoresis of MyRV1/S4ss dsRNAs from Δ*dcl2* and Δ*agl2* at 44 and 66 days a.c. Progression of DLSs between Δ*dcl2* and Δ*agl2* were compared. All virus-infected isolates of Δ*dcl2* developed DLSs even at 44 days a.c. While some virus-infected Δ*agl*2 subcultures showed very faint DLS bands (shown by white asterisks), DLSs were observed in all MyRV1/S4ss-infected Δ*agl2* isolates at 66 days a.c. with even lower intensity than those in Δ*dcl2*. Red bars in the Δ*dcl2* panel indicate the loss of cognate normal segments in agarose gel, while yellow arrowheads in the Δ*agl2* panel represent remnants of standard segments. Red asterisks show migration positions of S4.

While DLSs were detected in both MyRV1/S4ss-infected Δ*dcl2* and Δ*agl2*, a lower frequency of DLSs occurred in Δ*agl2* than in Δ*dcl2* at a certain time point after receiving MyRV1/S4ss (Figure 1). That is, most Δ*dcl2* derived isolates harbored DLSs 40 days a.c., but only some of the Δ*agl2* derived subcultures contained DLSs. To monitor the appearance of DLS-carrying isolates in these two hosts, a set of ten isolates per host was taken from the recipient side of the co-cultures used in the previous experiment shown in Figure 1. These were cultured sequentially in solid medium until specific time points: 44 days a.c., 66 days a.c. and 120 days a.c. (Figure 3 and data not shown). At 44 days a.c., all ten sub-isolates of Δ*dcl2* were confirmed to carry DLSs, although some subcultures contained DLSs showing weak band intensity, as expected from Figure 1. An increase in the number of DLS-carrying isolates in Δ*agl2* was observed only beyond 44 days a.c. After prolonged incubation (66 days a.c.), all subcultures carried DLSs in Δ*agl2*. At this time point, there was a difference between Δ*agl2* and Δ*dcl*2; while intact corresponding segments were completely replaced by DLSs in Δ*dcl*2-infected MyRV1/S4ss (see the red bars in lanes 4–9 for S1 and in lane 10 for S3 in Figure 3; Δ*dcl*2), they were still visible in Δ*agl2* with lower band intensity (see yellow arrowheads in Figure 3; Δ*agl2*). It was also noteworthy that two DLSs found in a single isolate at day 44 (Figure 3; Δ*dcl*2 lane 8) converged into one at 66 days a.c. Even at 120 days a.c. the gel profiles remained the same in the two fungal strains (data not shown). It should be noted that S10ss occasionally appeared below the S4ss band in some DLS-carrying strains after prolonged culture (data not shown). These results suggest that Δ*agl2* and Δ*dcl*2 differ at least in the rate at which DLSs develop and in the robustness of replacement of normal unaltered segments.

**Figure 4.**
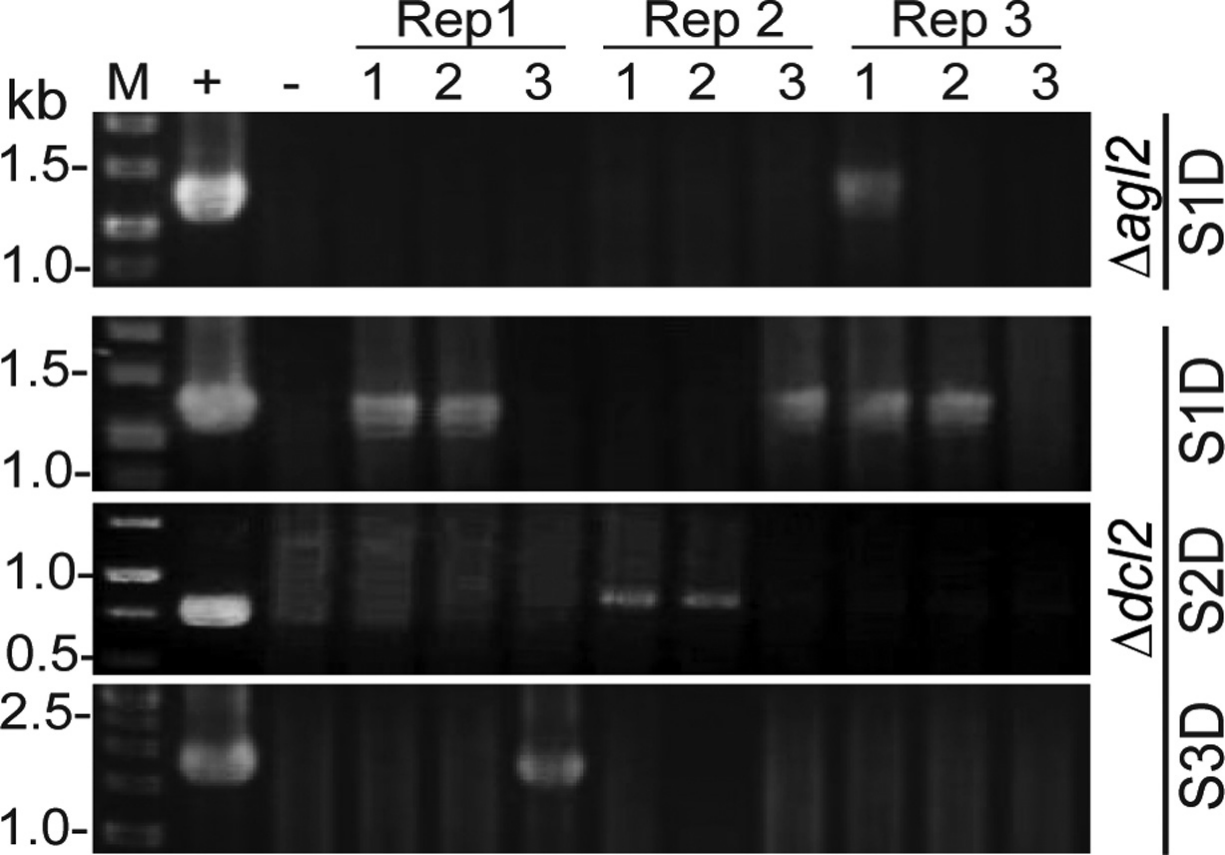
RT-PCR for specific detection of DLSs in Δ*dcl*2 and Δ*agl*2. A primer set was designed for back-to-back targeting of the jointed 5′- and 3′-terminal sequence of each DLS rearrangement (see Figure 2). When two-week-old culture materials were used, an approximately 1.2-kb PCR fragment of S1Da was detected in five Δ*dcl*2 subcultures and one Δ*agl*2 subculture. Results obtained with S2D and S3D-specific primers are shown in the middle and bottom panels, respectively. No PCR fragments were obtained in any Δ*agl*2 subculture using the S2Db- or S3Da-specific primer. Three samples per replicate (Rep) were assayed. The MyRV1/S4ss+S1Da, MyRV1/S4ss+S2Db and MyRV1/S4ss+S3Da dsRNAs were employed as internal controls for quantitative analysis (+). DLS-free WT MyRV1 was included as a negative control (−). M represents the DNA size marker.

To further confirm this difference in the rate of the appearance of DLSs in different host fungal strains, we utilized a highly sensitive method originally developed by (9) for detecting rotavirus rearrangements. This method allows for the detection of the junction of rearranged segments with duplications even at a ≥10 000:1 ratio of normal to rearranged molecules (the sensitivity threshold of RT-PCR). MyRV1/S4ss was horizontally transferred into Δ*dcl2* and Δ*agl2* via hyphal anastomosis, and a total of nine samples (three samples each from three independent co-cultures) per host were assayed by RT-PCR at six different time points (3, 5, 7, 10, 14 and 25 days a.c.; RT-PCR data are shown in Table 3 and in Figure 4). No PCR products were detected in either host strain until 7 days a.c. (Table 3). At 10 days a.c., only one sample from Δ*dcl2* was S1Da-positive, whereas no other samples provided PCR fragments. RT-PCR data for Δ*agl2* and Δ*dcl-2* infected with MyRV1/S4ss at 14 days a.c. are presented in Figure 4. Of nine Δ*dcl2* samples, five, two and one contained S1D, S2D and S3D, respectively, while only S1D was detected in a single Δ*agl2* sub-culture (Figure 4). Longer culture (25 days a.c.) resulted in an increased frequency of DLS generation in both strains (Table 3). As shown in Table 3, all types of DLSs (S1D–S3D) were formed in Δ*dcl2*. However, no S2D was found in Δ*agl2*. The dominance of S1D in Δ*dcl2* and the failure of S2D in Δ*agl2* were also repeatedly observed in a similar independent assay (Supplementary Table S2). It is also noteworthy that only one of the DLSs (S1D, S2D and S3D) was observed in certain single fungal subcultures. Consistent with the results of agarose gel electrophoresis from previous experiments (data not shown), no DLS-carrying isolate was detected in WT EP155 even by this sensitive method (Supplementary Table S2).

**Table 3.** Detection of duplicated large segments (DLSs) from Δ*agl2* and Δ*dcl2* by RT-PCR^a^

Sampling	Host^b^	Virus			No. of DLS (%)
Time		S1D	S2D	S3D	
7 days a.c.	Δ*agl2*	0	0	0	0 (0)
	Δ*dcl2*	0	0	0	0 (0)
10 days a.c.	Δ*agl2*	0	0	0	0 (0)
	Δ*dcl2*	1	0	0	1 (11)
14 days a.c.	Δ*agl2*	1	0	0	1 (11)
	Δ*dcl2*	5	2	1	8 (89)
25 days a.c.	Δ*agl2*	3	0	1	4 (44)
	Δ*dcl2*	6	2	1	9 (100)

^a^Wild-type EP155 infected with MyRV1/S4ss was fused with the indicated host strain and examined for DLS occurrence by RT-PCR.

^b^Δ*agl2* and Δ*dcl2* refer to the *Cryphonectria parasitica* mutant strains lacking *agl2* and *dcl2*.

These combined results indicate that while both Δ*dcl2 and* Δ*agl2* supported the occurrence of DLS, the two strains differed in both their generation rate and possession of intact segments.

### Duplicated large segments are transmitted horizontally and stably maintained in the absence but not in the presence of the *dcl2* gene

**Figure 5.**
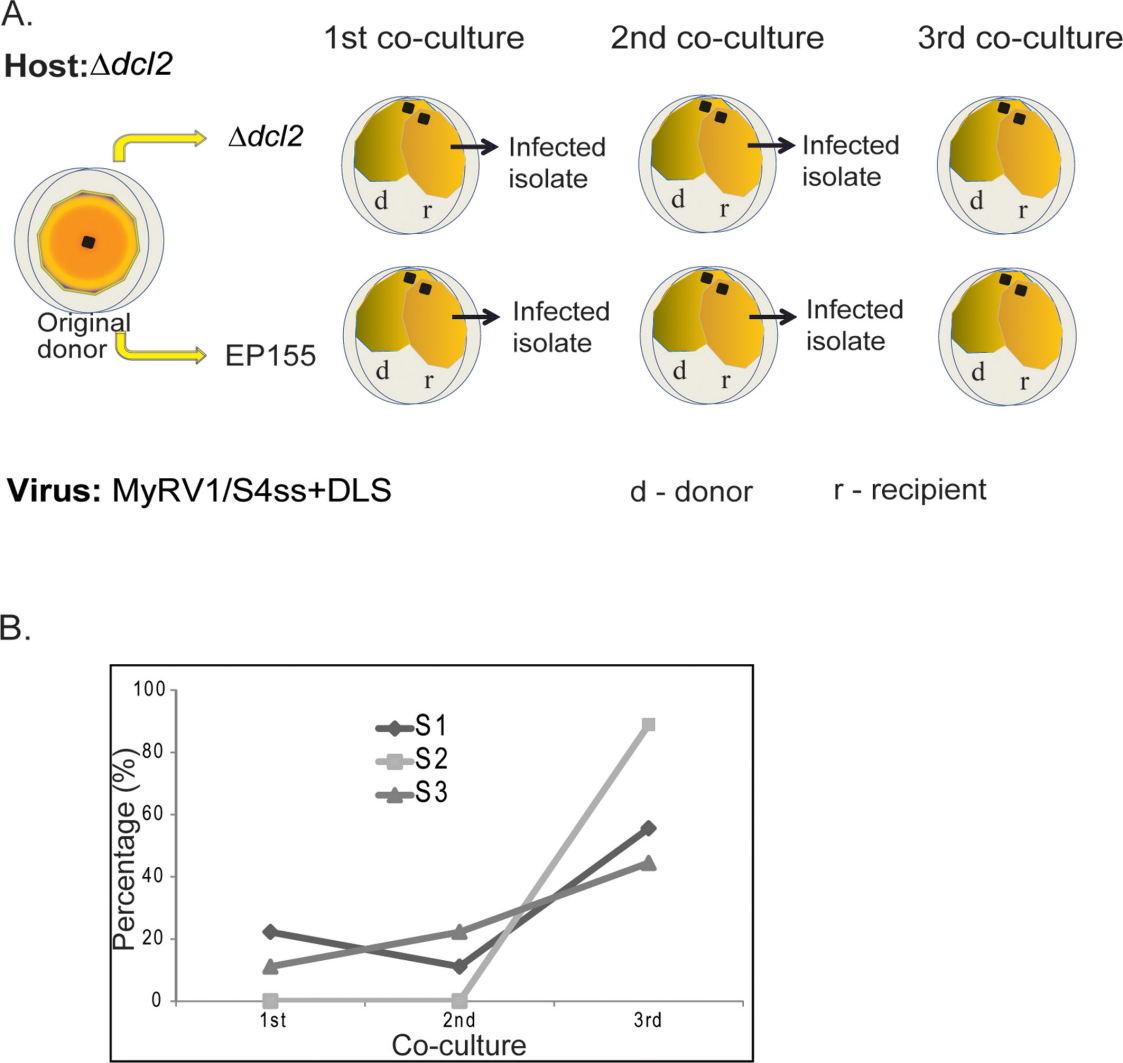
Horizontal transmission and stability of DLSs in Δ*dcl2* and EP155. (**A**) Schematic representation of the experiment flow. The original donor strain Δ*dcl2* infected with either of four virus strains WT MyRV1, MyRV1/S4ss+S1Da, MyRV1/S4ss+S2Db and MyRV1/S4ss+S3Da was co-cultured for four weeks with a recipient strain (r) EP155 or Δ*dcl2*. The resulting infected recipient from the first co-culture served as a virus donor (d) in the subsequent hyphal anastomosis experiments in respective host genotypes (second and third co-cultures). (**B**) Graph representing an increase of apparent revertants during the first to third co-culture.

Our initial observation showed that DLS-carrying isolates in Δ*dcl2* appeared stable when sub-cultured continuously for several weeks. We tested whether these DLSs are transferred and persist in the absence or presence of the *dcl2* gene. For this purpose, DLS-carrying MyRV1/S4ss strains were transferred in parallel to EP155 and Δ*dcl2* host backgrounds via hyphal anastomosis three times at four-week intervals, and examined for the presence of rearranged segments (Figure 5A). DLS-carrying isolates were grouped based on the lost cognate segment, termed MyRV1/S4ss+S1Da, MyRV1/S4ss+S2Db and MyRV1/S4ss+S3Da, and one of these representative isolates as a virus donor (Supplementary Figure S6) was used for transmission experiments.

Supplementary Figure S6 shows representative dsRNA gel profiles for WT MyRV1, DLS-carrying and non-carrying MyRV1/S4ss obtained after the first co-culture. All DLSs were transferred along with MyRV1/S4ss and maintained in Δ*dcl2* sub-isolates regardless of DLS type (Δ*dcl2*, left side). In contrast, the cognate segments of DLSs started to reappear or became dominant in some sub-isolates in the WT fungus (Supplementary Figure S6, EP155, right side). For example, S1Da (the rearranged segment) and S1 (the normal segment) co-existed in most of the EP155 sub-isolates infected with MyRV1/S4ss+S1Da and some sub-isolates lost S1D (lanes 3 and 6), while no S1 band was seen in any sub-isolates of Δ*dcl2* (see top panel). The ratio of S1Da to S1 varied among EP155 subcultures when examined based on visual inspection of the band intensity. A similar trend was observed for MyRV1/S4ss+S3Da (bottom panel). All sub-cultured derivatives of Δ*dcl2* contained S3Da but lacked S3 (invisible in the agarose gel), while the intact S3 appeared in WT EP155 with ratios different to that of S3Da. MyRV1/S4ss+S2Db showed a different maintenance profile at the first co-culture. In all subcultures, both S2D and S2 co-existed stably regardless of the host fungal strains at this time point (middle panel). Interestingly, some EP155 sub-isolates lost S2Db completely in the second and third co-cultures (apparent reversion) (Supplementary Figure S6). Similar fates were observed for S1Da and S3Da, but these were more frequently lost during further subcultures (data not shown).

The above results show that DLSs are stably maintained in the absence of the *dcl2* gene. However, upon reintroduction (first co-culture) of DLS-carrying isolates into WT host strains and during successive subcultures (subcultures 2 and 3), DLSs tended to become a minor population or are lost, and cognate segments were restored in reverse proportions. These findings clearly indicate that absence of DCL2 is required for stable maintenance of DLSs associated with MyRV1/S4ss.

### Duplicated large segments are encapsidated and transcribed

**Figure 6.**
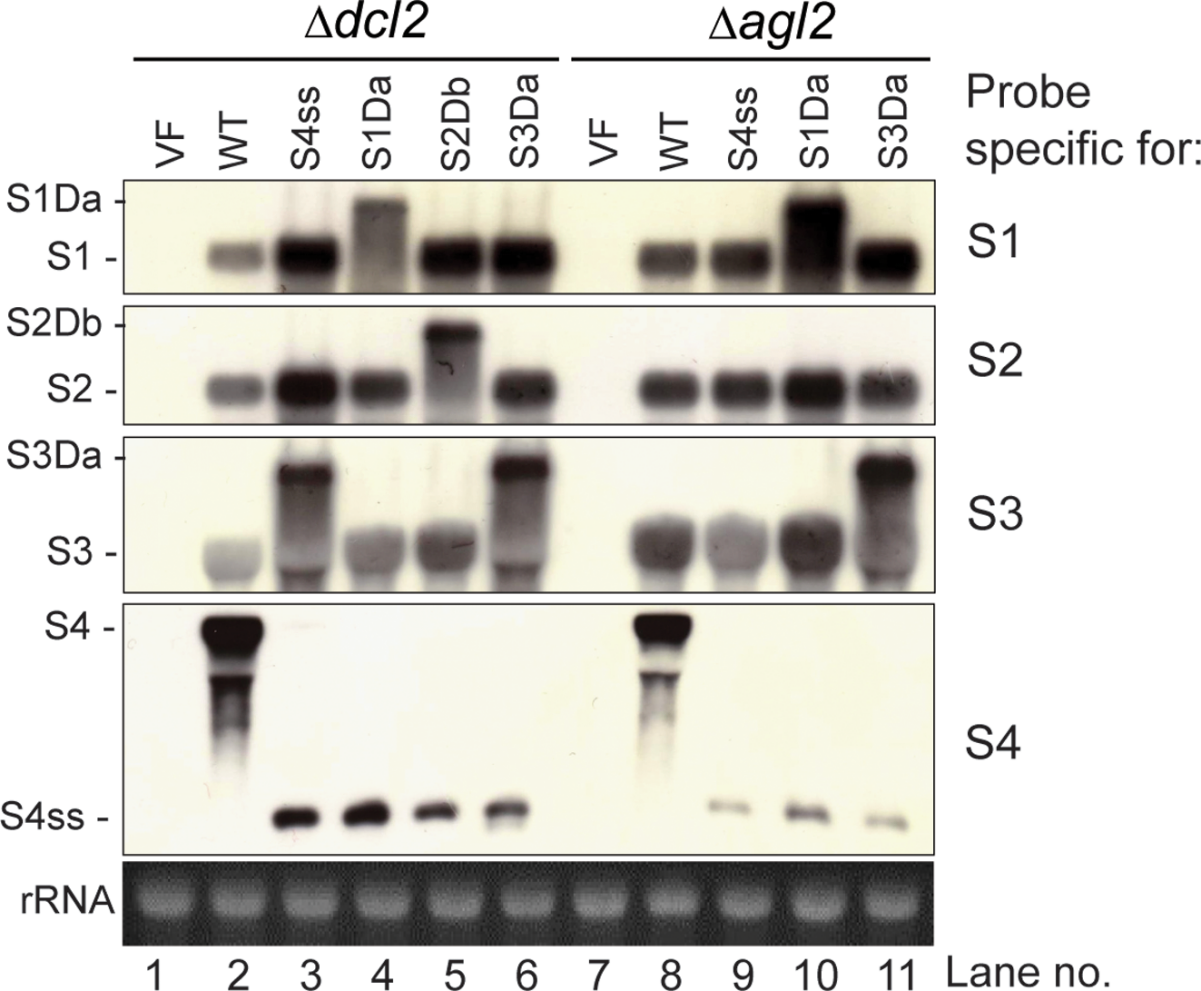
Northern blot analysis of DLS transcripts. SsRNA fractions from Δ*dcl2* and Δ*agl2* infected by either of the DLS-carrying (S1Da, S2Db, S3Da) and non-carrying (WT, S4ss) MyRV1 strains were analyzed by northern blotting, using S1 to S4 specific cDNA probes (see Material and Methods). The detection signals indicated that these DLSs were encapsidated and transcribed like normal cognate segments. Note that the MyRV1/S4ss-infected Δ*dcl2* produced S3D during sub-culture. All DLS-carrying fungal strains maintained S4ss and expected respective DLSs in these RNA silencing deficient hosts. The loading control was rRNA, as shown below the northern panels.

In order to test whether DLSs were encapsidated and transcribed, we performed northern analysis using representative strains of MyRV1 variants containing DLSs. Δ*dcl*2 and Δ*agl*2 were chosen as host backgrounds for this assay because they maintain DLSs stably. It was found that S1Da transcripts much larger than intact S1 transcripts were detectable in MyRV1/S4ss+S1Da by an S1-specific probe (Figure 6, lanes 4 and 10). The intact S1 transcripts were detectable for other MyRV1 variants. Similarly, an S2-specific probe allowed for the detection of much larger transcripts of S2Db in MyRV1/S4ss+S2Db (Figure 6, lane 5). S3Da transcripts were detected in MyRV1/S4ss+S3Da (Figure 6, lanes 6 and 11). Note that S3 of MyRV1/S4ss was rearranged into S3Da during culture in Δ*dcl*2 (Figure 6, lane 3), supporting the instant generation of DLS in this virus-host combination as was observed repeatedly in this study. As mentioned previously, MyRV1/S4ss+S2D was not obtained in Δ*agl*2 and therefore it was not included. In all fungal strains infected with MyRV1/S4ss variants, S4ss transcripts were observed as in the parental MyRV1/S4ss (Figure 6, S4ss), indicating the stable maintenance of S4ss in these mutant fungal strains. The WT virus showed intact S4 transcripts and no S4ss counterparts.

Given that reoviral transcription occurs in core particles using virion-associated RNA polymerase in the cytosol and viroplasm (42), these results show that all of the rearranged segments are encapsidated and transcribed properly.

## DISCUSSION

Relative to plant genes involved in RNA silencing, fungal counterparts are less numerous (43,44). There are four *rdr* genes, two *dcl* genes and four *agl* genes in *C. parasitica*. These numbers are smaller than those of some model plants, e.g. six, four, and ten and five, eight, and nineteen for *Arabidopsis thaliana* (45) and rice (46), respectively. Among these, only *dcl2* and *agl2* are known to play roles in the RNA silencing pathway in *C. parasitica* (23,37). While the *qde1* gene of a model filamentous fungus, *Neurospora crassa*, is required for transgene silencing (quelling) (41), deletion of its ortholog in *C. parasitica rdr1* or other *rdr* genes results in no phenotypic alteration associated with RNA silencing (25). We investigated the effects of deletion of *rdr1, agl2, dcl1* and *dcl2* (Table 1). Only RNA silencing-defective Δ*dcl2* and Δ*agl2* yielded a very high frequency of MyRV1/S4ss rearrangements (Figure 1, Table 2), and the two fungal strains showed different rates of DLS generation (Figures 3 and 4, Table 3). Therefore, there must be a certain link between RNA silencing deficiency and the appearance of rearrangements. Recently, RNA silencing has been shown to be required for, or to influence the recombination of positive strand RNA viruses. Nuss and coworkers (23,24) reported that DI-RNA (internally deleted forms) of the prototypic hypovirus CHV1-EP713 occurs only in WT *C. parasitica* strains, but not in the RNA silencing-deficient strains Δ*dcl2* and Δ*agl2*, the same fungal hosts as those used here. These findings led the authors to suggest that RNA silencing promotes recombination by a temperate switch, and generation and/or maintenance of DI-RNA (23,24). Bujarski and coworkers also showed that host plant mutants defective in DCLs alter the recombination profiles of a positive strand RNA plant virus, brome mosaic virus (47,48). The phenomenon described here is in stark contrast to these reported examples in terms of the involvement of RNA silencing in viral RNA recombination.

In plants, RNA silencing operates in a temperature-dependent manner and plants grown at higher temperatures show higher RNA silencing activities (49). This temperature dependence was not observed in MyRV1 DLS appearance. We examined the effects of temperature on DLS appearance in WT EP155, Δ*dcl*2 and Δ*agl*2 infected with MyRV1/S4ss. No significant difference was observed regardless of three different temperature settings, i.e. 20°C, 25°C or 30°C. MyRV1/S4ss+DLSs appeared only in Δ*dcl*2 and Δ*agl*2 within the range of frequencies shown in Table 2, but not in WT EP155. These results suggest that RNA silencing in WT host strains is sufficiently active at any tested temperature to inhibit the occurrence of DLSs.

The rearranged segments observed here are distinct from the rearrangements reported previously for reoviruses. The MyRV1/S4ss rearrangements found in RNA silencing-defective mutants have certain characteristics: (1) they all occur in the three largest segments, S1–S3, that encode structural proteins, i.e. RdRp (VP1), the major capsid protein (VP2) and the capping enzyme (VP3) (31,50) and (2) they contain almost complete duplications of the coding domain or entire genome segment in a head-to-tail orientation (Figure 2). Reported reovirus rearrangements are largely restricted to genomic segments encoding non-structural proteins (see (31) for a review). Exceptions include those found in plant reoviruses (51), rotaviruses (52), orbiviruses (53), and orthoreoviruses (54) where structural protein-encoding segments are internally deleted or partially duplicated. Almost complete duplications similar to S1Da and S3Da have never been reported for the three largest segments of any reovirus, although end-to-end template switching was reported to occur in S9 of a tissue culture-adapted strain of an orbivirus, epizootic hemorrhagic disease virus (53) and on artificially shortened templates *in vitro* by RdRps from positive strand RNA viruses (55). It has also been recently reported that in-frame extensions of MyRV1 S1 (S1L) to S3 (S3L) are generated in transformants with CHV1 p29 (31). The DLSs reported here differ from previously reported S1L–S3L in that the DLSs (except for S1Db and S2Db) exhibit complete genome duplication and have no alteration in their original coding capacity (Figure 2, S1Da and S3Da). A commonality is that the rearranged segments reported in this study and in the study by (31) contain other rearranged segments, i.e. S4ss and S10ss, respectively, the significance of which is discussed below.

The phenomenon described here is of interest from two perspectives: the capacity of inner core particles and *cis*- or *trans-*elements for *p*ackaging, *a*ssortment, *r*eplication, *t*ranscription and/or *t*ranslation (PARTT) (19). Inspection of Figure 6 suggests that DLSs are properly encapsidated and transcribed as efficiently as normal cognate segments, because reoviral core particles are believed to be the site of transcription. Based on studies of orthoreoviruses and rotaviruses, it is generally accepted that reoviral core particles can hold extra sequences corresponding to 10% of the entire genome size (56,57). MyRV1 carries a dsRNA genome of 11 segments (S1 to S11), ranging from 4127 to 732 bp; its total size is 23 433 bp (27). Considering the extension in S1D–S3D and the deletion in S4ss (1713 bp), the overall size increase in the variants with DLSs would fall well within the range set by the rule: 10.3% for S1Da, 9.9% for S1Db, 3.0% for S2Db and 6.6% for MyRV1/S3Da. PARTT is believed to reside in the terminal sequence portion that includes the 5′ and 3′ untranslated regions (UTRs) and possibly extends slightly to the coding domains (19,58). Recently, the ability to accommodate heterologous sequences was explored using reverse genetics of an orthoreovirus and a rotavirus (18,19). It was shown that secondary sequence structures affected the maintenance of altered sequences. In terms of the *cis*-element, all the DLSs reported here are expected to retain one or two sets of PARTT (Figure 2).

Extended segments of rotaviruses are known to be packaged preferentially over the corresponding parental segments such as segments 5, 7 and 11 (17,59,60). One explanation for this phenomenon is that a duplicated packaging signal may be present in the rearranged segments, thus enhancing their preferential packaging. In this regard, the rearranged segments of MyRV1 are different. Respective cognate standard segments gradually become dominant in the WT (EP155) fungal strain over extended segments of MyRV1 S1, S2, S3 and S6 (Figure 5) (30,31). However, this situation is reversed in RNA silencing-deficient (Δ*dcl2* and Δ*agl2*) or suppressed (Twtp29) fungal strains, i.e. MyRV1 DLSs are stably maintained in those host strains (Supplementary Figure S6).

Reovirus RNA synthesis is believed to occur within core particles or core-like particulates which engage the same number of RNA polymerase complexes as the genome segment number. It is anticipated that gene duplication events occur at the transcription step (multiple rounds of positive strand synthesis), not during replication (single round of negative strand synthesis) (61). A four-tunnel model was proposed for the key player in RNA synthesis, RdRp, based on 3D structures predicted by X-ray crystallography of reovirus and rotavirus RdRps (62–64). According to the model, two tunnels serve as intake gates for nucleoside triphosphates (NTPs) and negative strand RNA as the template in transcription, while the other two serve as exits for nascent positive strand RNA and the template RNA. In order for DLSs to occur during MyRV1 transcription, RdRp must retrieve the template RNA and continue the synthesis of a second copy before the nascent RNA disengages from its catalytic site. That is, the same template RNA reenters the same tunnel or is, although less likely, brought back in its single-stranded RNA form. The former case is reminiscent of the ‘loop’ model proposed by (61) for partial duplication of reoviral rearrangements.

It remains unknown as to how RNA silencing deficiency or suppression is related to MyRV1 rearrangements. Our previous studies showed the frequent occurrence of MyRV1 rearrangements in transformants with the CHV1 p29 (30,31). The present study suggests that the p29 activities generating MyRV1 rearrangements are a manifestation of its activity to suppress host RNA silencing (37,65), as discussed above. The observation that rearrangements occur in the RNA silencing-defective host strains in a manner specific to MyRV1/S4ss strongly suggests the relevance of the total genome size and rearrangement occurrence. Comparison of the rearrangement profiles of WT MyRV1 and MyRV1/S4ss occurring in Twtp29 supports this notion (Table 2, Experiment 1, Supplementary Figure S5, Table S2). DLSs were detected frequently and consistently only in MyRV1/S4ss-infected Twtp29, but infrequently in MyRV1-infected Twtp29 (31). Further support comes from previous observations of the rearrangements of S1L–S3L generated in Twtp29, which are always—or very frequently—associated with S10ss (31). It is of great interest to speculate that core particles with deleted segments might enhance further extension rearrangements to fill the core particle. This might stabilize them structurally and functionally by filling the core particles and ensuring proper placement of the enzyme complex responsible for RNA synthesis. For this purpose, the other segments S5-S11 might be too small, as the complete duplication of S5 would result in a size increase of 2.2 kbp, which is smaller than the smallest extension of 2453 bp (S2Da).

We previously offered two hypotheses to account for the CHV1 p29-mediated generation of MyRV1 rearrangements. One is that p29 is directly associated with MyRV1 replication based on physical *in vivo* interactions of CHV1 p29 and a MyRV1 non-structural protein VP9, as well as co-fractionation of p29 and the genomic dsRNAs (30). The other is that p29 alters the cellular environment favorable for the selection and/or maintenance of rearranged MyRV1 variants through an unknown mechanism. The generation of CHV1 p29-independent MyRV1/S4ss rearrangement and subsequent stable maintenance in RNA silencing-defective strains (Figures 1 and 3) confirms a scenario similar to the second possibility. RNA silencing might play a role in the suppression of MyRV1 genome rearrangements in which DCL2 is more effective in this capacity than AGL2. We cannot exclude the possibility that host factors other than DCL2 and AGL2, such as those in RNA decay pathways, are involved in the MyRV1 rearrangements, as is the case for the recombination of positive-strand RNA viruses (5). Another factor to consider is the possible beneficial effects of extension rearrangements for MyRV1/S4ss functionalization, stabilization and/or packaging as mentioned earlier. This complex interplay might determine the fate of DLSs in WT fungal and RNA silencing-deficient strains. However, a number of questions remain unanswered. Why were no rearrangements observed in WT MyRV1-infected Δ*dcl2* or Δ*agl2*? What is the molecular mechanism by which RNA silencing contributes to the suppression of MyRV1/S4ss genome rearrangement? What is the molecular basis of differences in the effectiveness of MyRV1 rearrangement generation and/or maintenance between Δ*dcl2* or Δ*agl2*, and how does it fit into the model recently proposed for RNA synthesis of a reovirus based on the structure of RdRp 3D (66)?

## SUPPLEMENTARY DATA

Supplementary Data are available at NAR Online.

SUPPLEMENTARY DATA
