# Supplementary material for: Mycoreovirus genome rearrangements associated with RNA silencing deficiency
Source: Nucleic Acids Res. 2015 Mar 23;43(7):3802–13. doi: 10.1093/nar/gkv239 (PMC4402544; doi:10.1093/nar/gkv239)
Supplement: SUPPLEMENTARY DATA [file supp_43_7_3802__index.html]

Mycoreovirus genome rearrangements associated with RNA silencing deficiency — Mycoreovirus genome rearrangements associated with RNA silencing deficiency — SUPPLEMENTARY DATA 

# Mycoreovirus genome rearrangements associated with RNA silencing deficiency

## SUPPLEMENTARY DATA

**Files in this Data Supplement:**

- SUPPLEMENTARY DATA
