# Supplementary material for: Mycoreovirus genome rearrangements associated with RNA silencing deficiency
Source: Nucleic Acids Res. 2015 Mar 23;43(7):3802–13. doi: 10.1093/nar/gkv239 (PMC4402544; doi:10.1093/nar/gkv239)
Supplement: SUPPLEMENTARY DATA [file supp_gkv239_nar-02982-a-2014-File011.pdf]

Supplementary data

Mycoreovirus genome rearrangements associated with RNA silencing deficiency

Ana Eusebio-Cope and Nobuhiro Suzuki*

Agrivirology Laboratory, Institute of Plant Sciences and Bioresources, Okayama University
Kurashiki, Okayama 710-0046, Japan

*Corresponding Author: Agrivirology Laboratory
Institute of Plant Sciences and Bioresources
Okayama University
Kurashiki, Okayama 710-0046
Japan
Tel. 81(86) 434-1230
Fax. 81(86) 434-1230
e-mail. nsuzuki@rib.okayama-u.ac.jp

Table S1. Oligodeoxynucleotide primers used in this study.

Primer name	Segment/ Gene	Sequence	map position	Used in
A	MyRV1 S1	GTCACGTTATGCTGATTC	3253-3270	RT-PCR for S1L detection
B	MyRV1 S1	GGATCTTGATTTCAGGTC	312-329	RT-PCR for S1L detection
C	MyRV1 S2	ATTCGCGCACATCGAAC	3170-3185	RT-PCR for S2L detection
D	MyRV1 S2	TTACCCATTCCAGTATGC	1430-1446	RT-PCR for S2L detection
E	MyRV1 S3	TCGTGTTGCAACTTGGAC	2343-2360	RT-PCR for S3L detection
F	MyRV1 S3	GCTAGGTACATAAATCGAC	788-806	RT-PCR for S3L detection
rdr1F1	*rdr1*	GAGCTCCACCCCTTA	916-930	PCR for probe preparation
rdr1R1	*rdr1*	ATGCGGTACGAGTTCT	2018-2033	PCR for probe preparation
hygF1	*hph*	CTGAACTCACCGCGACGTC TGTC	11-33	PCR for probe preparation
hygR1	*hph*	GTCGGTTTCCACTATCGGC GAGT	973-995	PCR for probe preparation
rdr1F2	*rdr1*	AGCCTGAATGTCCAATTC	1843-1860	RT-PCR for *rdr1*detection
rdr1R2	*rdr1*	CTCAGGATGATAGGTTGC'	2392-2409	RT-PCR for *rdr1*detection
actF1	*actin*	ATGTGCAAGGCCGGTTTCG	46-64	RT-PCR for detection of the actin gene
actR1	*actin*	TTAGAAGCACTTGCGGTGG	1110-1127	RT-PCR for detection of the actin gene

Table S2. Detection of duplicated large segments (DLSs) from different fungal hosts by RT-PCR*.

Sampling time	Host**	Rearranged segment			No. of DLS (%)
		S1D	S2D	S3D	
14 days a.c.	EP155	0	0	0	0 (0)
	Δ*agl2*	1	0	0	1 (11)
	Δ*dcl2*	4	1	2	7 (78)
	Twtp29	5	0	4	9 (100)
21 days a.c.	EP155	0	0	0	0 (0)
	Δ*agl2*	2	0	3	5 (56)
	Δ*dcl2*	6	1	2	9 (100)
	Twtp29	5	0	4	9 (100)
35 days a.c.	EP155	0	0	0	0 (0)
	Δ*agl2*	2	0	5	7 (78)
	Δ*dcl2*	7	1	1	9 (100)
	Twtp29	5	0	4	9 (100)

*These data sets were independently prepared of those shown in Table 3.

**EP155, Δ*agl2*, Δ*dcl2* and Twtp29 refer to the *Cryphonectria parasitica* wild-type strain, its mutant strains lacking *dcl2* and *agl2*, and the transgenic strain expressing p29 encoded by Cryphonectria hypovirus 1.

Legends to Supplemental Figures

Figure S1. Diagrammatic representation of the standard procedure for MyRV1 rearrangement assay. See Materials and Methods for details.

Figure S2. Disruption of the RNA dependent RNA polymerase 1 (*rdr*1) gene of *C. parasitica*. (A) Genomic organization of the wild-type (WT) *rdr1* allele encompassing 3,763-bp coding region with 4 exons (black boxes) interrupted by short introns (arrows). The *Apa* I and *Hin*d III restriction sites used for Southern analysis are indicated above the coding region. Below is the disruption construct containing the hygromycin phosphotransferase (*hph*) gene driven by the *C. parasitica* promoter glyceraldehyde-phosphate dehydrogenase gene (P*gpd*) and terminator (T*gpd*) that was used to replace *rdr*1 by homologous recombination. The plasmid contains the approximately 2-kb fragment *Sac* I-*Xba* I and *Sal* I-*Apa* I fragments flanking the *hph* cassette. (B) Southern blot analysis of *C. parasitica* WT strain EP155 and its *rdr*1-disruption mutant (Δ*rdr*1). Genomic DNA from both samples was digested with *Hin*d III and *Apa*1 having 1 or no cleavage sites in the *rdr1* gene, respectively. An *rdr*1-specific cDNA probe was prepared using a PCR DIG probe synthesis kit (Roche Diagnostics) with the position indicated in A (blue bar). Likewise, an *hph*-specific cDNA probe was prepared similarly (black bar). All restriction enzymes used do not cleave the *hph* gene expression cassette. (C) RT-PCR analysis of the *rdr1* transcripts. RNA samples from the WT EP155, Δ*dcl*1, Δ*dcl*2 and Δ*rdr*1 strains were used for RT-PCR targeting transcripts of the *rdr1* and actin genes. The *rdr1* transcripts were detectable in the WT and two mutants, Δ*dcl1* and Δ*dcl2*, but absent in Δ*rdr*1. Control PCR for the actin transcript was successful for all tested samples. The expected amplified region targeting the *rdr1* gene is shown in A (red bar). Primers used in this study are listed in Table S1.

Figure S3. Genome profiles of WT MyRV1 and MyRV1/S4ss. (A) Diagram of the genome segment gel profiles for the two viral strains. Blue bars refer to standard genome segments, while a rearranged segment S4ss is shown by a red bar. The red asterisks in this and subsequent panels indicate loss of S4 in the mutant strains, MyRV1/S4ss. Positions of

the normal segment and S4ss are denoted at the left of the panels. (B) Agarose gel electrophoresis of dsRNA from WT MyRV1 and MyRV1/S4ss used in this study. The S4 in MyRV1/S4ss is lost, and a short dsRNA termed S4ss appears. (C) Northern analysis of WT MyRV1 and MyRV1/S4ss. Single-stranded RNA fractions were prepared from the WT EP155 strains infected by WT MyRV1 and MyRV1/S4ss (used in B), respectively, and subjected to Northern analysis. Virus-free EP155 was analyzed in parallel. Ethidium bromide-stained rRNAs are shown as a loading control. cDNA probes specific to each of the 11 segments were used (see (1)). The results confirmed expected genome profiles for both viral strains and absence of any other types of genome alterations for the strains used in hyphal anastomosis.

Figure S4. Phenotypes of *C. parasitica* strains EP155, Δ*dcl2*, and Δ*agl2* infected and uninfected with MyRV1 (WT) and MyRV1/S4ss. Fungal colonies were grown on PDA for 7 days and photographed.

Figure S5. Agarose gel profiles of rearranged MyRV1 variants generated in Twtp29 infected WT MyRV1 and MyRV1/S4ss. DsRNA was isolated from a few representative fungal strains harboring rearranged segments. As reported previously by Sun and Suzuki (2), S10ss was mainly generated in MyRV1-infected Twtp29 strains, while the majority of rearrangements in MyRV1/S4ss-infected Twtp29 were DLSs. Arrowheads indicate S10ss, while the red bar denotes loss of S10. Note that lane 6 contains both S10 and S10ss.

Figure S6. DsRNA gel profiles of MyRV1/S4ss carrying duplicated large segments (DLSs) after the 1st subculture experiment. DsRNA fractions were isolated from 9 samples of each virus/host combination and electrophoresed in 1.0% agarose gel. The top panel shows a dsRNA gel pattern for MyRV1/S4ss+S1Da, while the second and third panels show those for MyRV1/S4ss+S2Db and MyRV1/S4ss+S3Da, respectively. DLSs of different sizes were readily evident in Δ*dcl2* infected by MyRV1/S4ss, but not in EP155. All of the DLSs persisted in Δ*dcl2*. In the WT EP155, reversion of some S1Da and S3Da to their cognate segments (yellow arrowheads) was observed. Most of these EP155-recipient isolates contained both DLSs and the revertant (cognate) form in a single fungal isolate with varying ratios. S2Db exhibited a similar retention profile in the host strains.

REFERENCES

1. Eusebio-Cope, A., Sun, L., Hillman, B.I. and Suzuki, N. (2010) Mycoreovirus 1 S4-coded protein is dispensable for viral replication but necessary for efficient vertical transmission and normal symptom induction. *Virology*, **397**, 399-408.
2. Sun, L. and Suzuki, N. (2008) Intragenic rearrangements of a mycoreovirus induced by the multifunctional protein p29 encoded by the prototypic hypovirus CHV1-EP713. *RNA*, **14**, 2557-2571.

Figure S1.

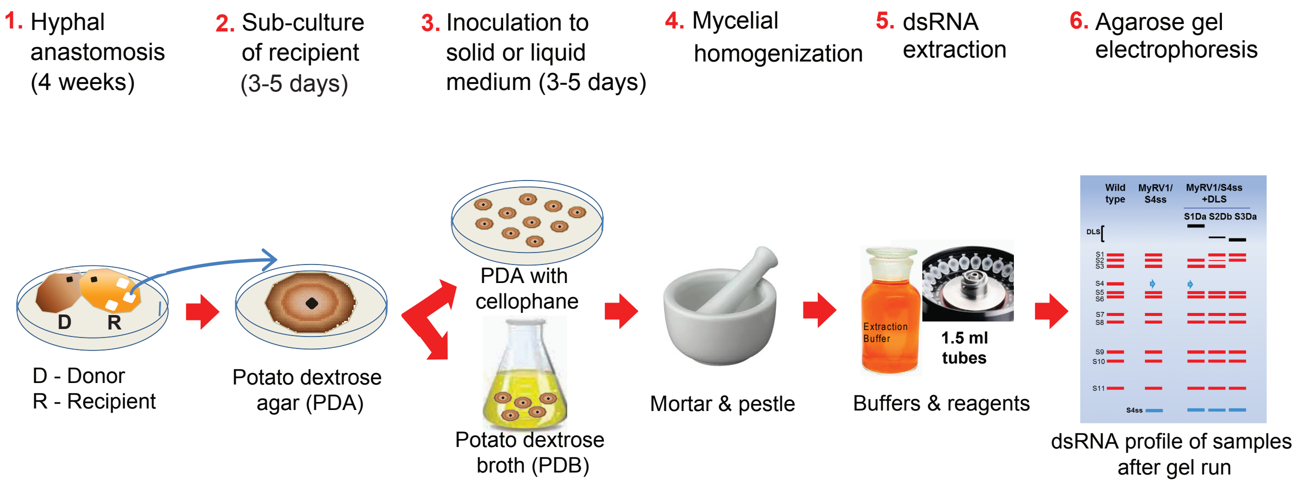

1. Hyphal anastomosis (4 weeks)
2. Sub-culture of recipient (3-5 days)
3. Inoculation to solid or liquid medium (3-5 days)
4. Mycelial homogenization
5. dsRNA extraction
6. Agarose gel electrophoresis
D
R
D - Donor
R - Recipient
Potato dextrose agar (PDA)
PDA with cellophane
Potato dextrose broth (PDB)
Mortar & pestle
Extraction Buffer
1.5 ml tubes
Buffers & reagents
Wild type
MyRV1/ S4ss
MyRV1/S4ss +DLS
S1Da S2Db S3Da
DLS
S4ss
dsRNA profile of samples after gel run

Figure S2.

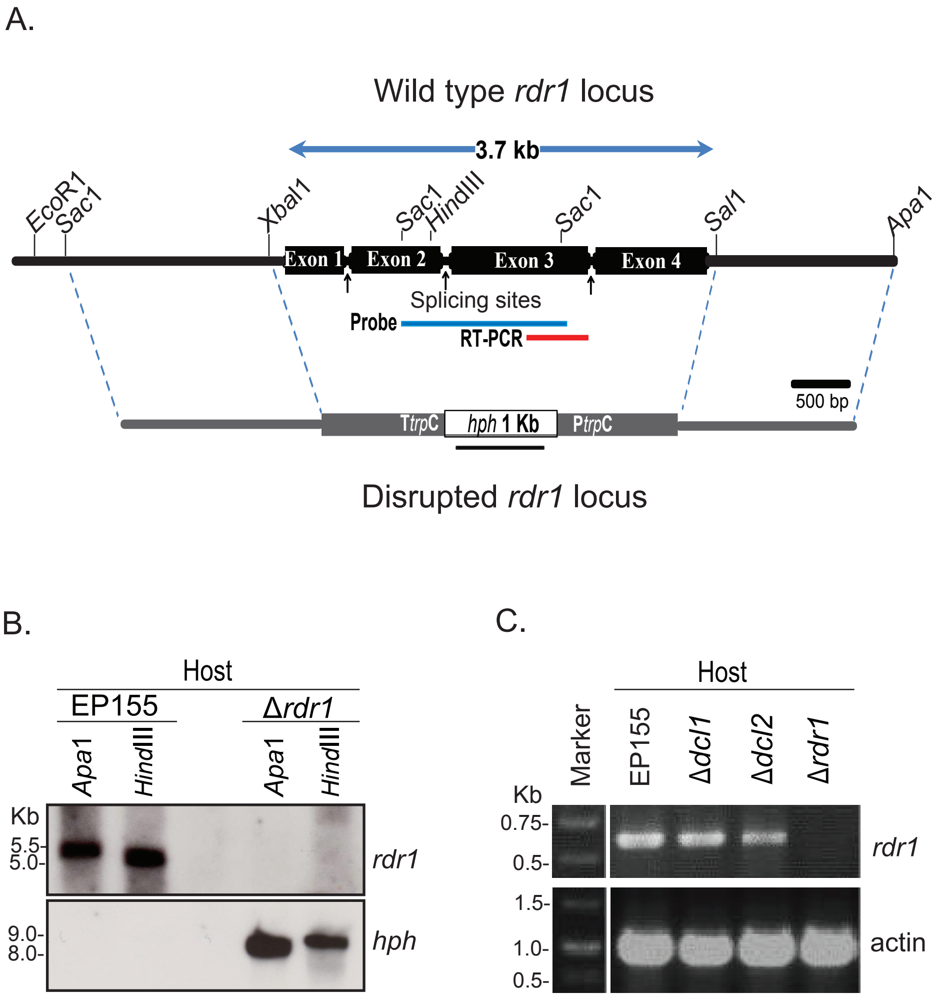

A.
Wild type rdr1 locus
3.7 kb
EcoR1
Sac1
Xba1
Sac1
HindIII
Sac1
Sal1
Apa1
Exon 1
Exon 2
Exon 3
Exon 4
Splicing sites
Probe
RT-PCR
500 bp
TtrpC
hph 1 Kb
PtrpC
Disrupted rdr1 locus
B.
Host
EP155
Δrdr1
Apa1
HindIII
Apa1
HindIII
Kb
5.5-
5.0-
rdr1
9.0-
8.0-
hph
C.
Host
Marker
EP155
Δdcl1
Δdcl2
Δrdr1
Kb
0.75-
0.5-
rdr1
1.5-
1.0-
0.5-
actin

Figure S3.

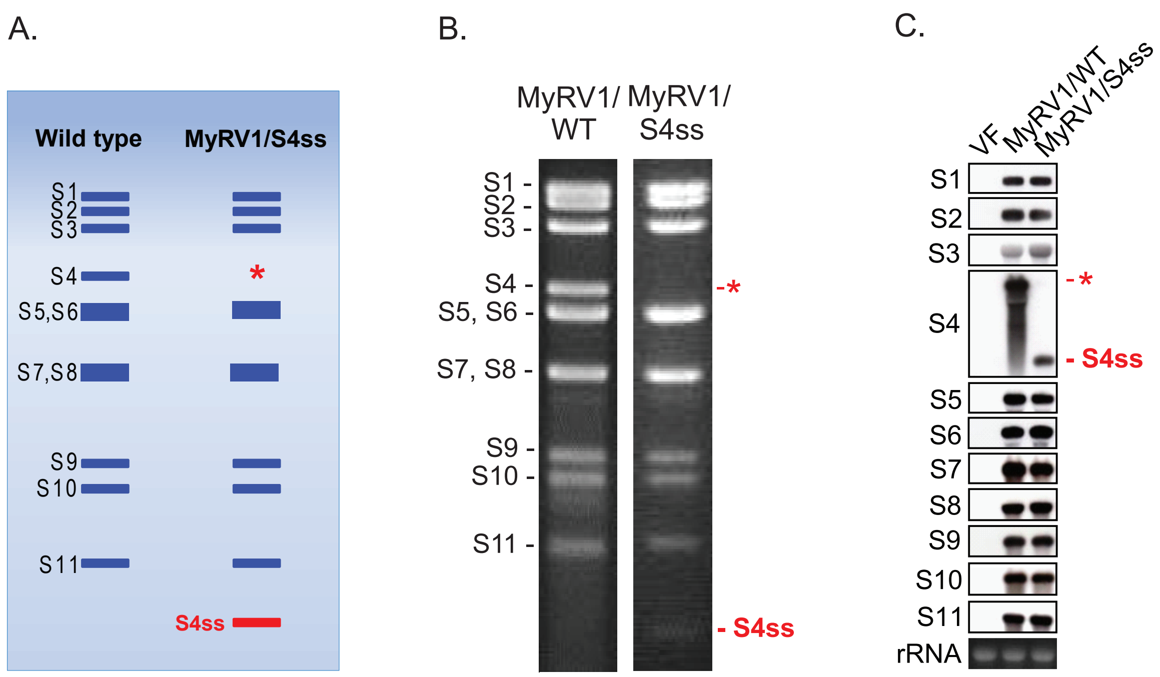
A.
Wild type
MyRV1/S4ss
S1
S2
S3
S4
*
S5,S6
S7,S8
S9
S10
S11
S4ss
B.
MyRV1/ WT
MyRV1/ S4ss
S1 -
S2 -
S3 -
S4 -
-*
S5, S6 -
S7, S8 -
S9 -
S10 -
S11 -
- S4ss
C.
VF
MyRV1/WT
MyRV1/S4ss
S1
S2
S3
S4
- *
- S4ss
S5
S6
S7
S8
S9
S10
S11
rRNA

Figure S4.

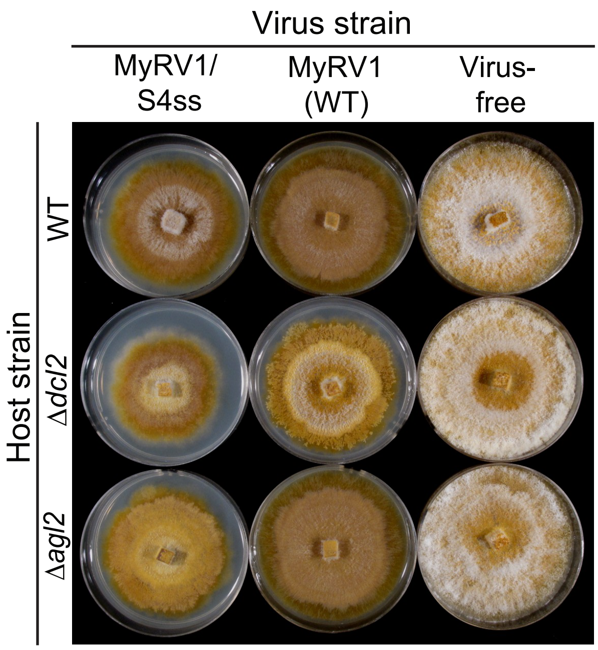
Virus strain
MyRV1/ S4ss
MyRV1 (WT)
Virus- free
Host strain
WT
Δdcl2
Δagl2

Figure S5.

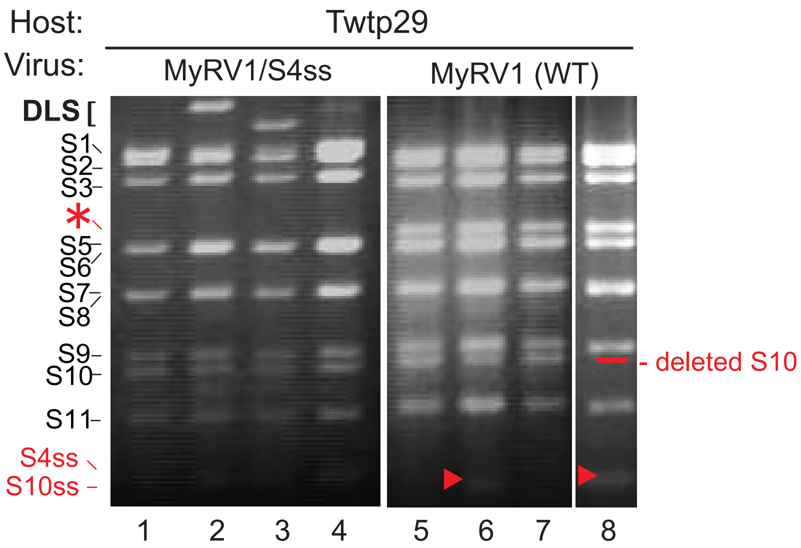

Host:
Twtp29
Virus:
MyRV1/S4ss
MyRV1 (WT)
DLS [
S1
S2
S3
*
S5
S6
S7
S8
S9
S10
S11
S4ss
S10ss
- deleted S10
1
2
3
4
5
6
7
8

Figure S6.

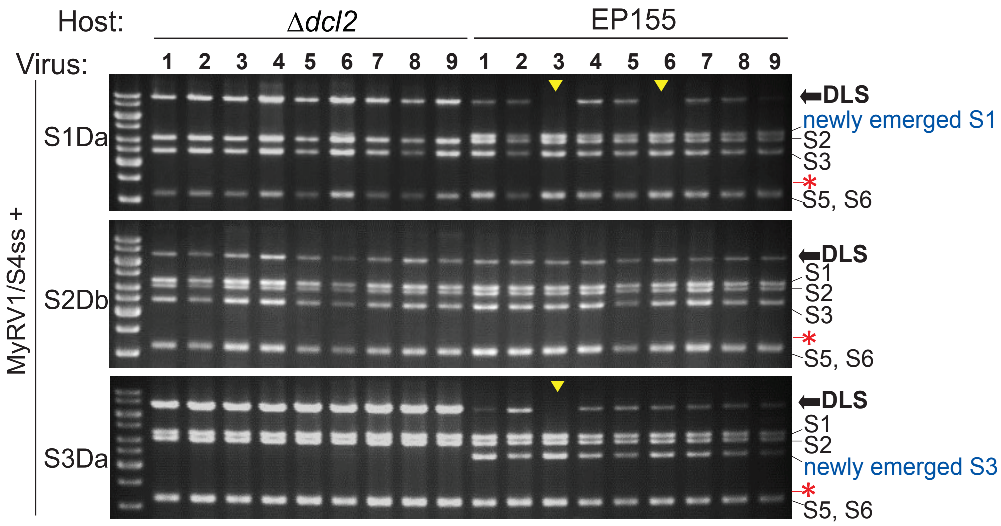

Host:
Δdcl2
EP155
Virus:
1 2 3 4 5 6 7 8 9 1 2 3 4 5 6 7 8 9
MyRV1/S4ss +
S1Da
S2Db
S3Da
DLS
newly emerged S1
S2
S3
*
S5, S6
DLS
S1
S2
S3
*
S5, S6
DLS
S1
S2
newly emerged S3
*
S5, S6
